# Autism spectrum disorder is associated with gut microbiota disorder in children

**DOI:** 10.1186/s12887-019-1896-6

**Published:** 2019-12-27

**Authors:** Hairong Sun, Zhong You, Libo Jia, Fang Wang

**Affiliations:** 1 Departemnt of Pediatrics, the Fifth People’s Hospital of Wuxi, 1215 Guangrui Road, Wuxi, 214145 Jiangsu Province China; 2 Departemnt of Pediatrics, the Fifth Affiliated Hospital of Jiangnan University, Wuxi, Jiangsu Province China; 3 Departemnt of Pediatrics, Huishan District Rehabilitation Hospital, Wuxi City, Jiangsu Province, China; 4 School of Medicine, Jiangnan University, 1800 Lihu Avenue, Wuxi, Jiangsu Province, 214122 China

**Keywords:** ASD, Gut microbiota, 16S sequencing, Operational taxonomic units, KEGG

## Abstract

**Background:**

The aim of this study was to evaluate the occurrence and clinical characteristics of autism spectrum disorder (ASD) associated to the stable state of the gut microbiota.

**Methods:**

A total of 9 children with ASD and 6 healthy children used as control were selected and feces samples were collected from all of them. The 16S gene ribosomal RNA sequencing was used to analyze the difference in gut microbiota between healthy control children and ASD patients.

**Results:**

The results of 16S sequencing based on operational taxonomic units (OTUs) analysis showed that the ASD group and the healthy control (HC) group had a large difference in the abundance of microbiota at the level of family, genus and species. The abundance of Bacteroidales and Selenomonadales was significantly lower in the ASD group than in the HC group (*p* = 0.0110 and *p* = 0.0076, respectively). The abundance of Ruminococcaceae in the ASD group was higher than that in the HC group (*p* = 0.0285), while the amount of Prevotellaceae was significantly lower in the ASD group than in the HC group (*p* = 0.0111). The Tax4Fun analysis based on Kyoto Encyclopaedia of Genes and Genomes (KEGG) data indicated differentially expressed functional pathway between the ASD group and healthy control group associated to the nervous system, environmental information processing and cellular processing.

**Conclusions:**

The abundance of gut microbiota in the ASD group is different from that in the healthy control children. These differences affect the biological function of the host. These results suggest that a disorder in the gut microbiota may be associated, at least in part, with ASD in children.

## Background

In recent years, autism spectrum disorder (ASD) has been associated with the microbial gut composition and its different pathophysiological effects (such as toxic substances associated with intestinal immune responses, increased neuronal alpha-synuclein delivery or increased permeability of the systemic inflammation) [1–3]. Children with ASD are prone to dysregulation in the intestinal microbiota due to environmental and behavioral factors and because of the imperfect development of the immune system [4], especially the nervous immune system [5], thus, disorders of the gut microbiota may have a greater impact on children with ASD [6–8]. According to the above evidence, the gut microbiota from fecal samples of ASDs children and healthy control (HC) children was analyzed. Our results showed a significant difference in the microbial community composition between ASDs and HCs. Thus, children’s targeted improvement of intestinal microbiota might be helpful in improving the health of ASD children.

## Methods

### Study design and patient selection

This study selected children with autism who were included according to the corresponding symptoms of typical autism of the International Classification of Disease (ICD-11 version) issued by the WHO (World Health Organization) [9], accompanied by speech disorders, communication difficulties, and intelligence. Typical state such as abnormal development. A total of 9 ASD patients aged 3–12 years, receiving rehabilitation in the rehabilitation center for children with autism at the Department of Autism Rehabilitation, Wuxi Huishan District Rehabilitation Hospital, China, from December 2018 to April 2019 were enrolled in our study and considered as the ASD group. The gastrointestinal function of these children were normal (without symptoms such as vomiting, diarrhea, and/or constipation, just to mention some) in the period of time from 3 months before the collection of the stool samples to the date of collection. In addition, 6 healthy children with an age matching the one of the patients were selected from kindergartens in the same area, and were considered as the HC group. 16S gene ribosomal RNA sequencing was performed on fecal samples of all the 15 children.

### RNA sequencing and data analysis

Feces were collected from each subject, placed in a sterile stool container, frozen immediately in liquid nitrogen and stored at − 80 °C. Since fecal samples differed in their collection dates, total bacterial DNA was extracted from fecal samples within 1 month using the QIAamp DNA Stool Mini Kit (Qiagen, Valencia, California) according to the manufacturer’s protocol with minor modifications. The V3-V4 region of the 16S ribosomal RNA (rRNA) gene was amplified and sequenced on the Illumina MiSeq platform (Illumina, San Diego, California) in multiple runs, pooling together all 15 samples using a 2 × 250 bp paired-end protocol, according to the manufacturer’s instructions. Raw reads from the microbiota sequencing were analyzed using Pandaseq, processed through the QIIME (version 1.8.0), clustered into operational taxonomic units (OTUs) at 97% identity level and taxonomically assigned via Ribosomal Database Project classifier against the Greengenes database (release 13.5; http://greengenes.secondgenome.com) [10]. The KEGG pathways database was used to predict differences in bacterial biochemical pathways between the ASD group and HC group. A *p* value <0.001 was considered statistically significant.

### Statistical analysis

Statistical evaluation of differences in alpha-diversity measures was performed by a nonparametric Monte Carloifferences in bacterial biochemical pathwaysadonis” donistical evaluation of di “vegan” (https://cran.r-project.org/web/packages/vegan/index.html) was used to determine statistical separation of the microbiota profiles in terms of beta diversity and predictive analysis. Other analyses were performed with the software SPSS 20.0 (Chicago), including two-tailed t-test and Pearson Chi-square test. *P* values < 0.05 were considered statistically significant.

## Results

### Microbial composition in ASD and HC

**Fig. 1:**
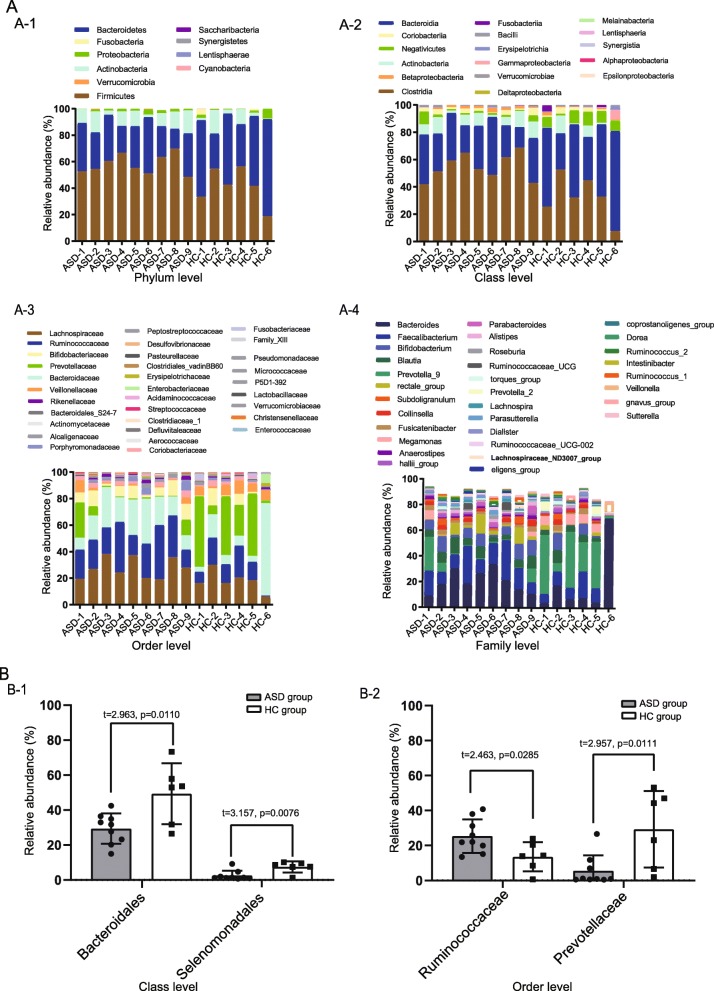
A Expression abundance of gut microbiota. Abundance diversity of gut microbiota in ASD group and HC group at the Phylum level, (A-1); Abundance diversity of gut microbiota in ASD group and HC group at the Class level, (A-2); Abundance diversity of gut microbiota in ASD group and HC group at the Order level, (A-3); Abundance diversity of gut microbiota in ASD group and HC group at the Family level, (A-4). B Abundance of ASD group and HC group. Differences in expression at Class level, Bacteroidales and Selenomonadales, (B-1); Differences in expression at Order level, Ruminococcaceae and Prevotellaceae, (B-2). *P* < 0.05*,*P* < 0.01**

**Fig. 2:**
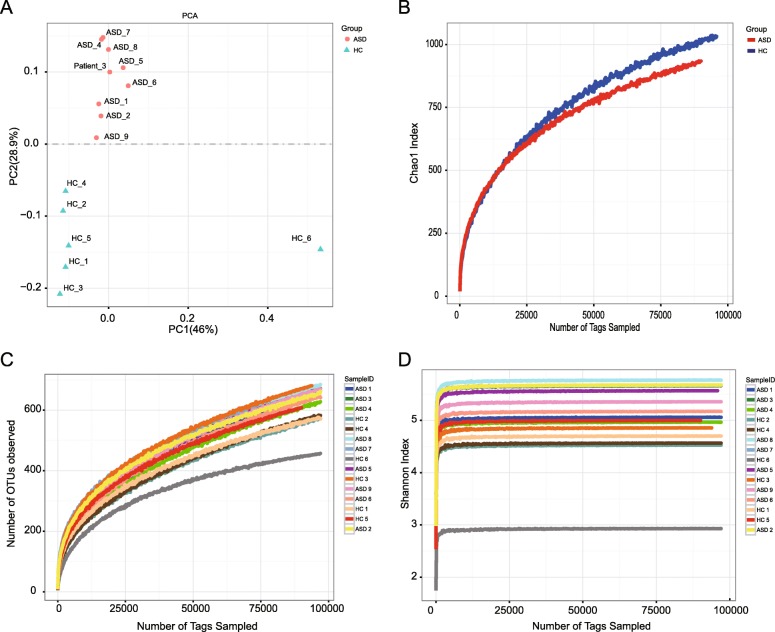
**a** Principal component analysis of ASD group and HC group. **b** Chao1 index curves of ASD group and HC group. **c** The number of OTUs observed curves of ASD group and HC group. **d** Shannon index curves of ASD group and HC group

**Fig. 3:**
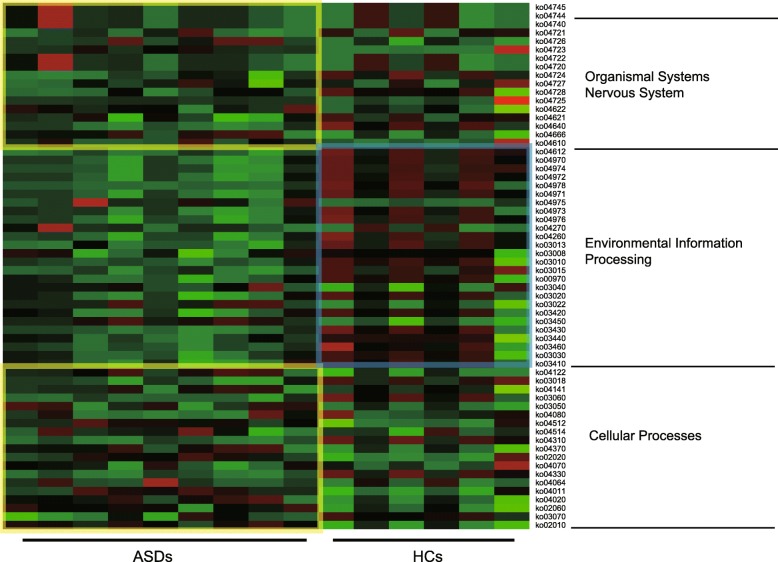
The predicted KEGG categories abundance of the expression in ASD group and HC group

In our study, a total of 15 fecal samples from children were collected by us, 9 ASDs and 6 HCs. The basic features of the 15 children included in our study are shown in Table 1. The microbial composition at the Phylum level (relative abundance >5% in the 15 samples) is shown in Fig. 1A-1. Firmicutes was the most abundant genus in all samples. The microbial composition at the Class level (relative abundance >1% in the 15 samples) is shown in Fig. 1A-2. The level of Bacteroidales in the ASD group (29.291 ± 8.689) was significantly less than that in the HC group (49.310 ± 17.384) (t = 2.963, *p* = 0.0110). The abundance of Selenomonadales in the ASD group (0.069 ± 0.031) was significantly less than that in the HC group (7.423 ± 3.186) (t = 3.157, *p* = 0.0076) (Fig. 1B-1). The microbiota at the Order level is shown in Fig. 1A-3. The average abundance was 1.0%. The abundance of 31 species was above the average in all 15 samples (Fig. 1A-4). The average abundance of Ruminococcaceae was higher in the ASD group (25.378 ± 9.562) compared to that in the HC group (13.561 ± 8.313), (t = 2.463, *p* = 0.0285). The average abundance of Prevotellaceae (5.540 ± 8.836) in the ASD group was lower than that in the HC group (29.313 ± 21.913), (t = 2.957, *p* = 0.0111), as show in Fig. 1B-2. The microbial composition at the Family level (relative abundance >1% in the 15 samples) is shown in Fig. 1A-4. Bacteroides and Prevotella_9 were the most abundant genus across all samples.

**Table 1:** Characteristics of 15 children

No	Sample	Age (months)	Confirmed age (months)	Cesarean section	Breastfeeding
1	ASD 1	≥ 60	≥ 30	Present	Absent
2	ASD 2	< 60	≥ 30	Present	Absent
3	ASD 3	≥ 60	< 30	Present	Present
4	ASD 4	≥ 60	< 30	Absent	Absent
5	ASD 5	≥ 60	≥ 30	Absent	Absent
6	ASD 6	< 60	< 30	Present	Present
7	ASD 7	≥ 60	≥ 30	Present	Present
8	ASD 8	≥ 60	< 30	Present	Present
9	ASD 9	≥ 60	< 30	Absent	Absent
10	HC 1	≥ 60	–	Present	Present
11	HC 2	< 60	–	Absent	Present
12	HC 3	< 60	–	Absent	Absent
13	HC 4	≥ 60	–	Absent	Present
14	HC 5	< 60	–	Absent	Absent
15	HC 6	< 60	–	Present	Present

### Microbiota and clinical features of ASD children

Table 2 shows the baseline data associated to the ASD and HC children enrolled in this study. The correlation between ASD and HC and gender, cesarean section and breastfeeding was further analyzed. A significant difference in gender, cesarean section and breastfeeding was not obtained between the two groups because of the small sample size. However, based on the differential analysis of gut microbiota of ASD and HC group, striking results were obtained. As regard the principal component analysis, ASD and HC children are distinguished in different quadrants, and they have distinct gut microbiota composition (Fig. 2A). The same result was obtained with the Tags Sampled analysis. The Chao1 index of ASD and HC group also showed the characteristics of two different biomes (Fig. 2B). In other alpha diversity analyses, the analysis of the number of OTUs and the Shannon Index also reflected the differences between ASD and HC children (Fig. 2C and Fig. 2D, respectively).

**Table 2:** Baseline parameters of 15 children

Baseline parameters	Inclusion study		*P value*
	ASD (*N* = 9)	HC (*N* = 6)	
Gender			0.292
Male	8 (88.9%)	4 (66.7%)	
Female	1 (11.1%)	2 (33.3%)	
Cesarean section			0.205
Present	6 (66.7%)	2 (33.3%)	
Absent	4 (33.3%)	4 (66.7%)	
Breastfeeding			0.398
> =10 months	4 (44.4)	6 (66.7)	
< 10 months	5 (55.6)	2 (33.3)

### Predictive microbiota functional profiling

The functional contribution of the bacteria in the ASD and HC group was predicted based on OTUs using the Tax4Fun package in R software. A total of 53 KEGG orthology were found across all samples, mainly belonging to the pathway Organismal Systems, Environmental Information Processing, and Cellular processes. The most abundant functional pathways are presented in Fig. 3. Cluster analysis of the KEGG pathway revealed that the gut microbes differentially expressed in the ASD group are mostly representing the Organismal Systems pathway. In the analysis of this heat map, the sub-classification of the Organismal Systems difference between the ASD group and the HC group was found located in the Nervous System Pathway. Similarly, the Environmental Information pathway was mostly represented in the ADS group and was also significantly lower in the ADS group than in the HC group.

## Discussion

The alteration in the profile of gut microbiota was investigated in a population of patients with ASD and correspondent HCs by applying a robust statistical approach, adjusting for potential confounders. Although our results confirm that ASD was characterized by several changes in microbiota composition when compared with HCs, the results showed that the analysis might be affected by a variety of confounding factors, in which the severity of the disease itself has a certain impact on the results [11]. Our study was more concerned on the effects of gut microbiota imbalance on the nervous system and cognitive function of ASD and HC. ASD children abundance of Bacteroidales and Selenomonadales was significantly lower than that in normal children. The difference in the level of gut microbiota at the class level is remarkably significant for the host, which has also been reported in other studies related to the relationship between neurological lesions and gut microbiota [12]. The dysregulation of Bacteroidales and Selenomonadales affects intestinal secretion and in turn affects brain function through the vagal response [13]. The role of Bacteroidales is to maintain the immune regulation of intestinal mucosal cells such as CD4 + T cells, CD8+ T cells and TLR responses, and they may also affect the development of host neurological function [14]. In addition, the within-group analysis according to ASD clinical features suggests the potential role of microbial composition (in particular Ruminococcaceae and Prevotellaceae) and the differential effects of the gut microbiota that further affect the children.

In the intestinal microbiota biodiversity analysis, the concentration trend in HCs was significantly different from the one in the ADS children. In the intestinal microbiota biodiversity analysis in the ASD children, the trend of HC concentration is significantly different, and in the alpha diversity analysis based on the biological microbiota, the trend of intestinal microbiota diversity reflecting ASD children is different from HCs [15, 16]. Because children’s nervous system and immune system are not yet well developed, factors such as cesarean section and breastfeeding were consider because they have a significant impact on the microbial community of infants and young children, and these differences affect children’s development [17, 18]. However, the baseline parameters of the children included in this study are referred to a small sample size and, thus, the results were not significant. However, in combination with the biological function prediction of KEGG, it was remarkable to notice that the imbalance of intestinal microbiota led to the inhibition of ASD nervous system and environmental information processes, in addition to the changes of Cellular Processes, which explained the influence of microbiota imbalance to some extent. Since the gut microbiota is controlled by the brain - gut axis, it affects the cognitive abilities of children with ASD and their responses to environmental information.

Due to our small sample size, the relationship between ASD and HC baseline parameters and cesarean section, breastfeeding and other factors, as well as the occurrence of ASD and gut microbiota imbalance, did not significantly differ. Autism is also a disease that is affected by multiple genes and environmental factors. However, the impact of genetic factors is not considered in our present research. Thus, further studies with a larger sample and prospective follow-up study is needed. However, our results demonstrated that it might be possible to improve the state of the nervous system development in patients with ASD by altering the food intake of children with ASD, thus interfering in a positive manner with the composition of the gut microbiota [19–21].

## Conclusion

The gut microbiota composition between ASD and HC group was significantly different. These differences might affect the development of the nervous system and the biology of the environmental information. Improvement and control of Ruminococcaceae for Bacteroidales, Selenomonadales and Prevotellaceae in ASDs children might improve the status of ASD.

## Data Availability

The datasets used and/or analysed during the current study are available from the corresponding author on a validated request.

## Abbreviations

ASD: Autism spectrum disorder
HC: Healthy control
OTU: Operational taxonomic unit
